# 

**Published:** 1885-12

**Authors:** 


ORIGINAL COMMUNICATIONS:

Diseases of the Period of Dentition. W. C. Barrett........ .. ............ ........ 623
Materials for Finishing and Polishing Fillings. C. F. W. Bodecker.......................... 640
Editorial Responsibility. Geo. H. Cushing... 643

REPORTS OF SOCIETY MEETINGS:

American Dental Association.................. 644
The Section of Oral and Dental Surgery in the International Medical Congress.... ......... 655
New York Odontological Society........ ..... 661

EDITORIAL:

To Junior Dentists. No. III.................. 664
Editors and Editing........................... 668
The Dental Section of the International Congress ........................................ 671
Close of the Volume. .. ...................... 673

CURRENT NEWS AND OPINION:

Obituary........................................ 674
Circular Letter to Members of Section VII. "Physiology and Etiology" of the American Dental Association ........ ............... 675
Connecticut Valley Dental Society............ 677
A Good Investment.............................. 677
A Treatise on Diphtheria.......... .. ......... 677
American Academy of Dental Science.. ...... 678
A Want Supplied................................. 678
Massachusetts Dental Society................. 678

